# Supplementary material for: High pathogenicity avian influenza A (H5N1) clade 2.3.4.4b virus infection in a captive Tibetan black bear (Ursus thibetanus): investigations based on paraffin-embedded tissues, France, 2022
Source: Microbiol Spectr. 2024 Feb 2;12(3):e03736-23. doi: 10.1128/spectrum.03736-23 (PMC10913436; doi:10.1128/spectrum.03736-23)
Supplement: Fig. S1 to S4 — Additional illustrations of histopathological findings, viral antigen and RNA distribution, and maximum likelihood phylogenetic trees performed on viral segments other than HA. [file spectrum.03736-23-s0001.pdf]

Supplementary Figures 1 to 3

Additional illustrations of histopathological findings, viral antigen and RNA distribution in tissues from a Tibetan black bear and a black-headed gull naturally-infected with H5N1 clade 2.3.4.4b HPAIV.

Supplementary figure 1. Additional illustrations of histopathological findings, viral antigen and RNA distribution in tissues from a Tibetan black bear naturally-infected with H5N1 clade 2.3.4.4b HPAIV.

a. Heart: the myocardium appears within normal limits (H&E stain). b. Heart: there is mild non-specific background staining with no detection of viral antigen (IHC). c. Heart: sparse viral RNA detection is observed within the myocardial interstitium (insert), M gene RNAscope ISH. d. Spleen: thrombosis is observed within the splenic parenchyma (arrowhead) (H&E stain). e. Spleen: IHC reveals mild to moderate non-specific background staining with no significant detection of viral antigen. f. Spleen: rare detection of viral RNA is observed within the red pulp (M gene RNAscope ISH). g. Kidney: there is congestion and hemorrhages within the renal interstitium (H&E stain). h. Kidney: sparse viral antigen detection is observed within glomerular tufts (IHC). i. Kidney: no viral RNA is detected (RNAscope ISH). j. Liver: the hepatic parenchyma is multifocally replaced by nodular suppurative foci (arrowhead) (H&E stain). k. Liver: No viral antigen detection is observed (IHC).). l. Liver: no viral RNA is detected (RNAscope ISH). Scale bars: 50µm (d-l) 100 µm, 200 µm (a-c).

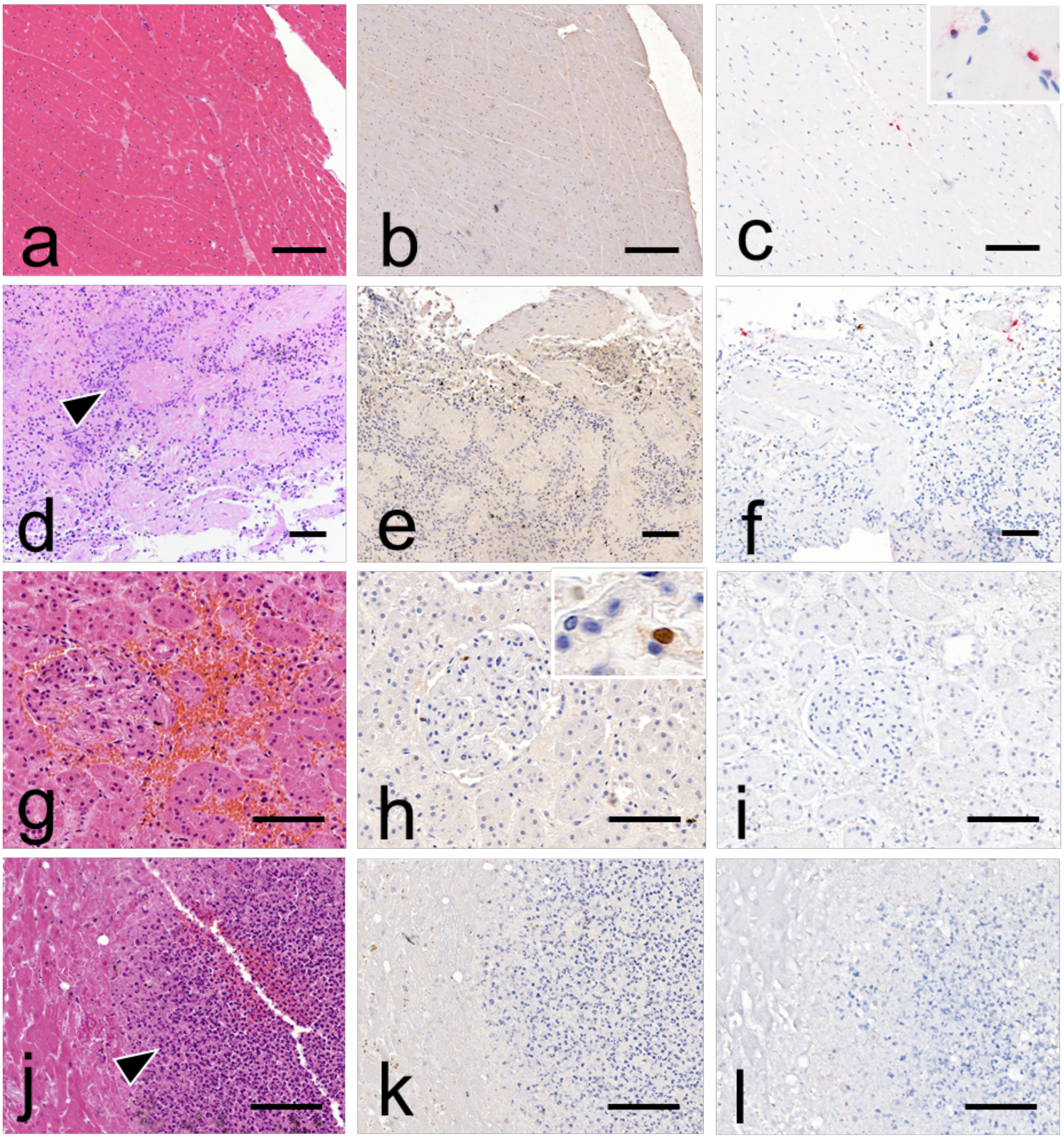

Supplementary figure 2. Additional illustrations of histopathological findings, viral antigenic and RNA distributions in tissues from a black-headed gull naturally infected with H5N1 clade 2.3.4.4b HPAIV.

a. Optic Lobe (Central Nervous System, CNS): mild congestion is observed at a subgross view (H&E stain). b. Optic Lobe (CNS): extensive viral antigen detection involving neurons, neuropil, and glial cells (IHC). c. Optic Lobe (CNS): extensive viral RNA detection also involving neurons, glial cells and, to a lesser extent, neuropil (RNAscope ISH). d. Kidney: segmental multifocal epithelial necrosis involving renal tubules (arrowhead) (H&E stain). e. Kidney: multifocal segmental positive viral antigen detection within tubular nephrocytes (IHC). f. Kidney: multifocal segmental positive viral RNA detection within tubular nephrocytes (RNAscope ISH). g. Prerenal Nervous Ganglion: within normal limits (arrowhead) (H&E). h. Prerenal Nervous Ganglion: no viral antigen detection (IHC). i. Prerenal Nervous Ganglion: positive viral RNA detection within several neurons (RNAscope ISH). j. Intestine: the mucosa exhibits epithelial sloughing (arrowhead), while the outer layers appear within normal limits (H&E stain). k. Intestine: no viral antigen is observed (IHC). l. Intestine: no viral RNA is detected (RNAscope ISH). Scale bars: 100 µm (d-l), 200 µm (a-c).

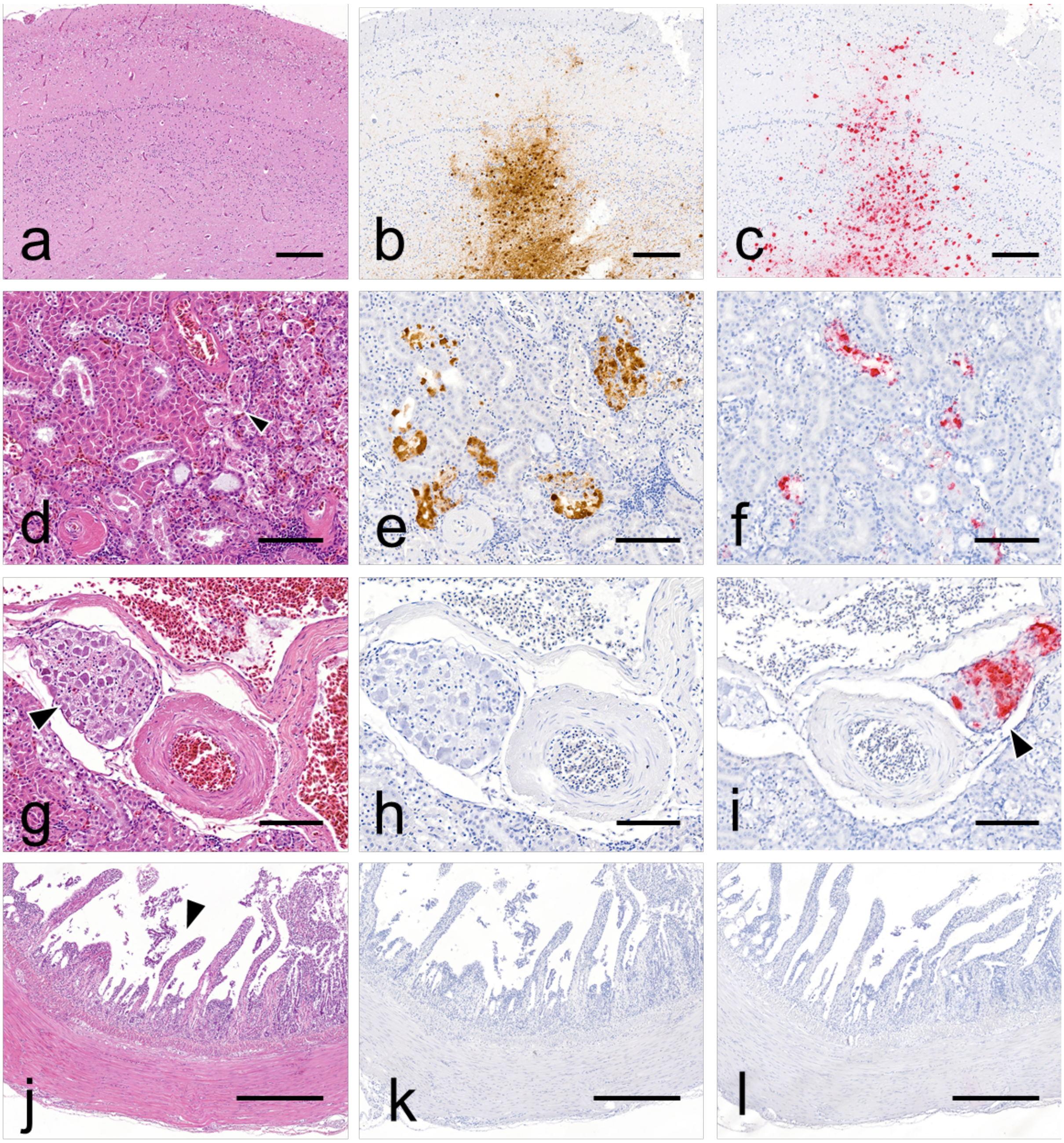

Supplementary figure 3. Additional illustrations of histopathological findings, viral antigen distribution in tissues from a black-headed gull naturally-infected with H5N1 clade 2.3.4.4b HPAIV.

a. Thyroid Gland: degeneration and necrosis of thyroid follicles is observed (arrowhead) (H&E stain). b. Thyroid Gland: viral antigen detection involving both lining and sloughed epithelial cells of a single follicle (IHC). c. Heart: mild focal myocardial degeneration and necrosis (arrowhead) (H&E stain). d. Heart: multifocal viral antigen detection within cardiomyocytes (IHC). e. Lung: pulmonary lobules appear within normal limits (H&E stain). f. Lung: multifocal viral antigen detection within the capillary bed (IHC). g. Spleen and Pancreas (splenic Lobe): the splenic parenchyma (s) is within normal limits, while diffuse pancreatic necrosis is observed (p) (H&E stain). h. Spleen and Pancreas (Splenic Lobe): no viral antigen is observed in the spleen, while extensive positivity is present within the necrotic pancreatic parenchyma, presumably in exocrine acinar cells. Scale bars: 100 µm (a-f), 400 µm (g, h).

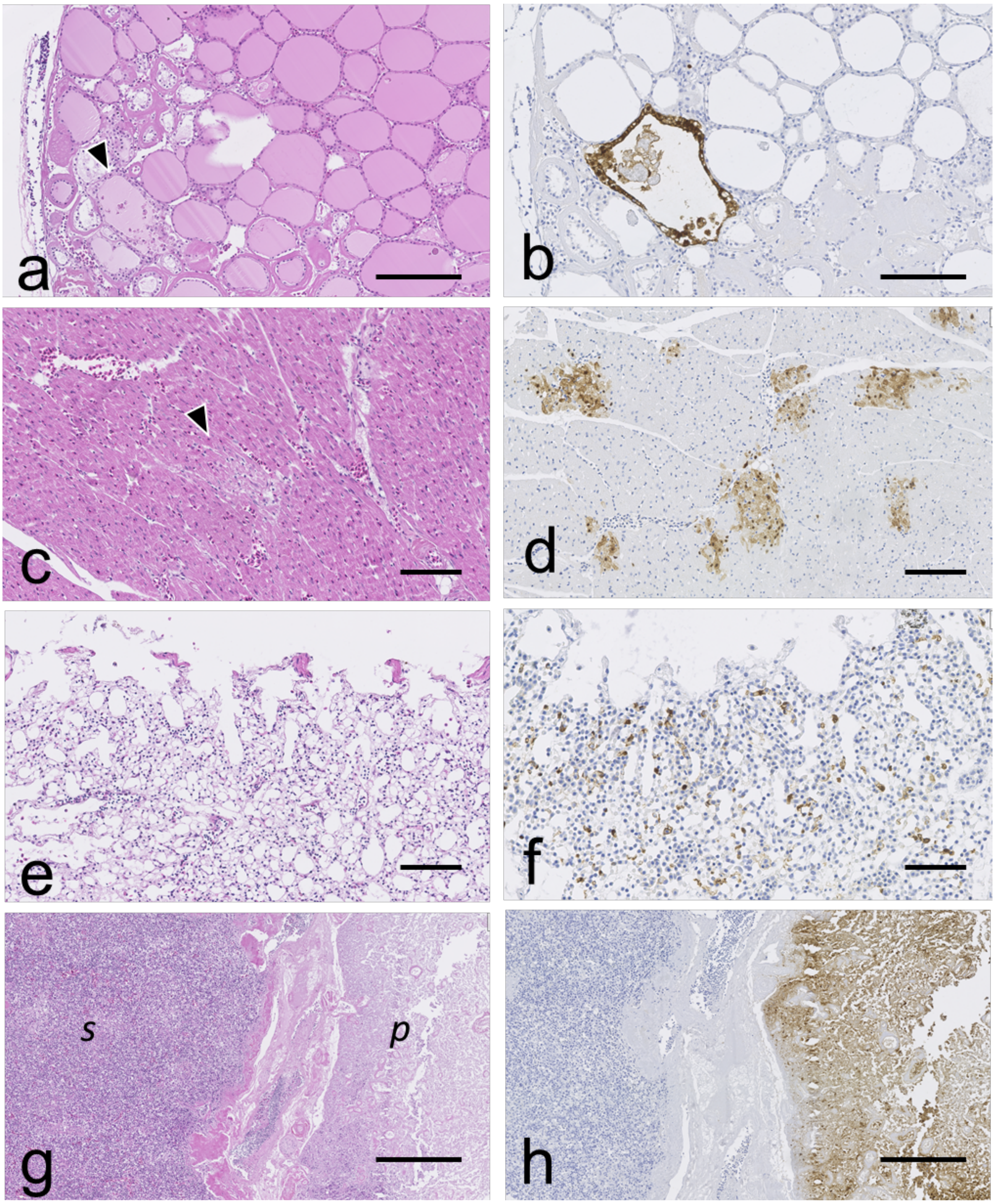

Supplementary Figure 4

Maximum likelihood phylogenetic trees performed on viral segments other than HA. Bear and gull-derived sequences are labelled in red and blue respectively. Bootstrap support values > 90 are shown at branches. Scale bar: number of nucleotide substitution per site.

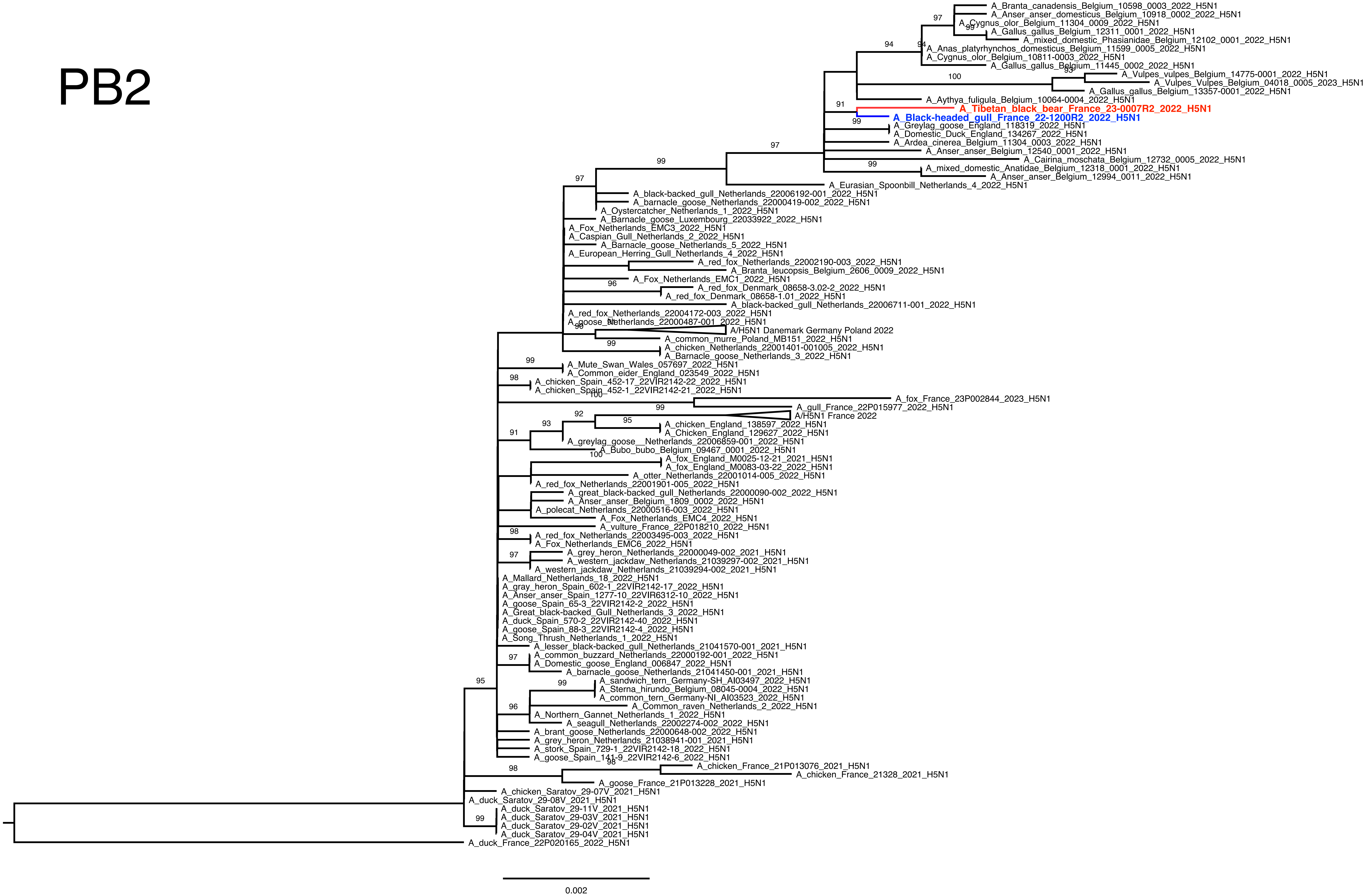
PB2
A_Branta_canadensis_Belgium_10598_0003_2022_H5N1
A_Anser_anser_domesticus_Belgium_10918_0002_2022_H5N1
A_Cygnus_olor_Belgium_11304_0009_2022_H5N1
A_Gallus_gallus_Belgium_12311_0001_2022_H5N1
A_mixed_domestic_Phasianidae_Belgium_12102_0001_2022_H5N1
A_Anas_platyrhynchos_domesticus_Belgium_11599_0005_2022_H5N1
A_Cygnus_olor_Belgium_10811-0003_2022_H5N1
A_Gallus_gallus_Belgium_11445_0002_2022_H5N1
A_Vulpes_vulpes_Belgium_14775-0001_2022_H5N1
A_Vulpes_Vulpes_Belgium_04018_0005_2023_H5N1
A_Gallus_gallus_Belgium_13357-0001_2022_H5N1
A_Aythya_fuligula_Belgium_10064-0004_2022_H5N1
A_Tibetan_black_bear_France_23-0007R2_2022_H5N1
A_Black-headed_gull_France_22-1200R2_2022_H5N1
A_Greylag_goose_England_118319_2022_H5N1
A_Domestic_Duck_England_134267_2022_H5N1
A_Ardea_cinerea_Belgium_11304_0003_2022_H5N1
A_Anser_anser_Belgium_12540_0001_2022_H5N1
A_Cairina_moschata_Belgium_12732_0005_2022_H5N1
A_mixed_domestic_Anatidae_Belgium_12318_0001_2022_H5N1
A_Anser_anser_Belgium_12994_0011_2022_H5N1
A_Eurasian_Spoonbill_Netherlands_4_2022_H5N1
A_black-backed_gull_Netherlands_22006192-001_2022_H5N1
A_barnacle_goose_Netherlands_22000419-002_2022_H5N1
A_Oystercatcher_Netherlands_1_2022_H5N1
A_Barnacle_goose_Luxembourg_22033922_2022_H5N1
A_Fox_Netherlands_EMC3_2022_H5N1
A_Caspian_Gull_Netherlands_2_2022_H5N1
A_Barnacle_goose_Netherlands_5_2022_H5N1
A_European_Herring_Gull_Netherlands_4_2022_H5N1
A_red_fox_Netherlands_22002190-003_2022_H5N1
A_Branta_leucopsis_Belgium_2606_0009_2022_H5N1
A_Fox_Netherlands_EMC1_2022_H5N1
A_red_fox_Denmark_08658-3.02-2_2022_H5N1
A_red_fox_Denmark_08658-1.01_2022_H5N1
A_black-backed_gull_Netherlands_22006711-001_2022_H5N1
A_red_fox_Netherlands_22004172-003_2022_H5N1
A_goose_Netherlands_22000487-001_2022_H5N1
A/H5N1 Danemark Germany Poland 2022
A_common_murre_Poland_MB151_2022_H5N1
A_chicken_Netherlands_22001401-001005_2022_H5N1
A_Barnacle_goose_Netherlands_3_2022_H5N1
A_Mute_Swan_Wales_057697_2022_H5N1
A_Common_eider_England_023549_2022_H5N1
A_chicken_Spain_452-17_22VIR2142-22_2022_H5N1
A_chicken_Spain_452-1_22VIR2142-21_2022_H5N1
A_fox_France_23P002844_2023_H5N1
A_gull_France_22P015977_2022_H5N1
A/H5N1 France 2022
A_chicken_England_138597_2022_H5N1
A_Chicken_England_129627_2022_H5N1
A_greylag_goose__Netherlands_22006859-001_2022_H5N1
A_Bubo_bubo_Belgium_09467_0001_2022_H5N1
A_fox_England_M0025-12-21_2021_H5N1
A_fox_England_M0083-03-22_2022_H5N1
A_otter_Netherlands_22001014-005_2022_H5N1
A_red_fox_Netherlands_22001901-005_2022_H5N1
A_great_black-backed_gull_Netherlands_22000090-002_2022_H5N1
A_Anser_anser_Belgium_1809_0002_2022_H5N1
A_polecat_Netherlands_22000516-003_2022_H5N1
A_Fox_Netherlands_EMC4_2022_H5N1
A_vulture_France_22P018210_2022_H5N1
A_red_fox_Netherlands_22003495-003_2022_H5N1
A_Fox_Netherlands_EMC6_2022_H5N1
A_grey_heron_Netherlands_22000049-002_2021_H5N1
A_western_jackdaw_Netherlands_21039297-002_2021_H5N1
A_western_jackdaw_Netherlands_21039294-002_2021_H5N1
A_Mallard_Netherlands_18_2022_H5N1
A_gray_heron_Spain_602-1_22VIR2142-17_2022_H5N1
A_Anser_anser_Spain_1277-10_22VIR6312-10_2022_H5N1
A_goose_Spain_65-3_22VIR2142-2_2022_H5N1
A_Great_black-backed_Gull_Netherlands_3_2022_H5N1
A_duck_Spain_570-2_22VIR2142-40_2022_H5N1
A_goose_Spain_88-3_22VIR2142-4_2022_H5N1
A_Song_Thrush_Netherlands_1_2022_H5N1
A_lesser_black-backed_gull_Netherlands_21041570-001_2021_H5N1
A_common_buzzard_Netherlands_22000192-001_2022_H5N1
A_Domestic_goose_England_006847_2022_H5N1
A_barnacle_goose_Netherlands_21041450-001_2021_H5N1
A_sandwich_tern_Germany-SH_AI03497_2022_H5N1
A_Sterna_hirundo_Belgium_08045-0004_2022_H5N1
A_common_tern_Germany-NI_AI03523_2022_H5N1
A_Common_raven_Netherlands_2_2022_H5N1
A_Northern_Gannet_Netherlands_1_2022_H5N1
A_seagull_Netherlands_22002274-002_2022_H5N1
A_brant_goose_Netherlands_22000648-002_2022_H5N1
A_grey_heron_Netherlands_21038941-001_2021_H5N1
A_stork_Spain_729-1_22VIR2142-18_2022_H5N1
A_goose_Spain_141-9_22VIR2142-6_2022_H5N1
A_chicken_France_21P013076_2021_H5N1
A_chicken_France_21328_2021_H5N1
A_goose_France_21P013228_2021_H5N1
A_chicken_Saratov_29-07V_2021_H5N1
A_duck_Saratov_29-08V_2021_H5N1
A_duck_Saratov_29-11V_2021_H5N1
A_duck_Saratov_29-03V_2021_H5N1
A_duck_Saratov_29-02V_2021_H5N1
A_duck_Saratov_29-04V_2021_H5N1
A_duck_France_22P020165_2022_H5N1
0.002

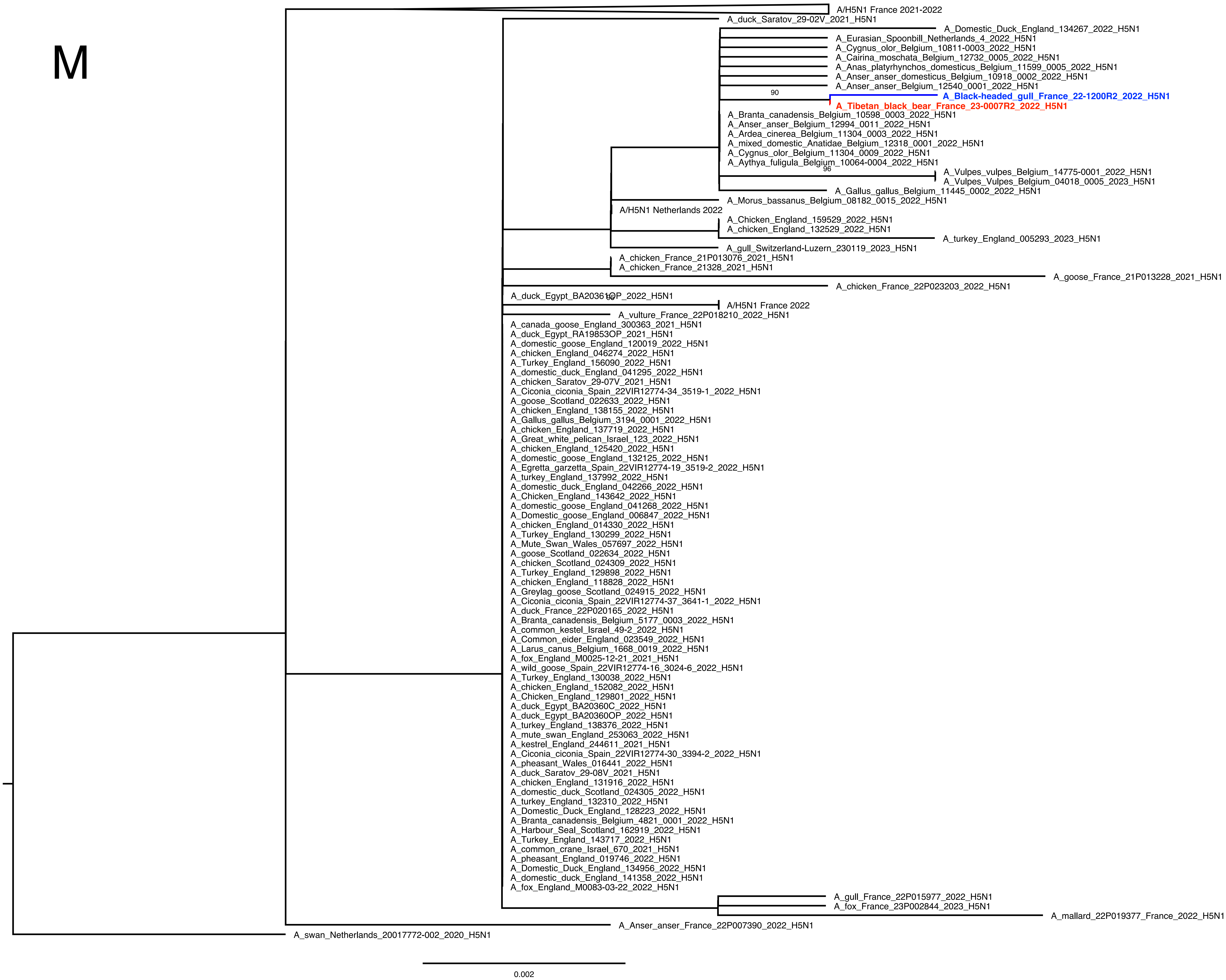
M
A/H5N1 France 2021-2022
A_duck_Saratov_29-02V_2021_H5N1
A_Domestic_Duck_England_134267_2022_H5N1
A_Eurasian_Spoonbill_Netherlands_4_2022_H5N1
A_Cygnus_olor_Belgium_10811-0003_2022_H5N1
A_Cairina_moschata_Belgium_12732_0005_2022_H5N1
A_Anas_platyrhynchos_domesticus_Belgium_11599_0005_2022_H5N1
A_Anser_anser_domesticus_Belgium_10918_0002_2022_H5N1
A_Anser_anser_Belgium_12540_0001_2022_H5N1
90
A_Black-headed_gull_France_22-1200R2_2022_H5N1
A_Tibetan_black_bear_France_23-0007R2_2022_H5N1
A_Branta_canadensis_Belgium_10598_0003_2022_H5N1
A_Anser_anser_Belgium_12994_0011_2022_H5N1
A_Ardea_cinerea_Belgium_11304_0003_2022_H5N1
A_mixed_domestic_Anatidae_Belgium_12318_0001_2022_H5N1
A_Cygnus_olor_Belgium_11304_0009_2022_H5N1
A_Aythya_fuligula_Belgium_10064-0004_2022_H5N1
96
A_Vulpes_vulpes_Belgium_14775-0001_2022_H5N1
A_Vulpes_Vulpes_Belgium_04018_0005_2023_H5N1
A_Gallus_gallus_Belgium_11445_0002_2022_H5N1
A_Morus_bassanus_Belgium_08182_0015_2022_H5N1
A/H5N1 Netherlands 2022
A_Chicken_England_159529_2022_H5N1
A_chicken_England_132529_2022_H5N1
A_turkey_England_005293_2023_H5N1
A_gull_Switzerland-Luzern_230119_2023_H5N1
A_chicken_France_21P013076_2021_H5N1
A_chicken_France_21328_2021_H5N1
A_goose_France_21P013228_2021_H5N1
A_chicken_France_22P023203_2022_H5N1
A_duck_Egypt_BA20361OP_2022_H5N1
95
A/H5N1 France 2022
A_vulture_France_22P018210_2022_H5N1
A_canada_goose_England_300363_2021_H5N1
A_duck_Egypt_RA19853OP_2021_H5N1
A_domestic_goose_England_120019_2022_H5N1
A_chicken_England_046274_2022_H5N1
A_Turkey_England_156090_2022_H5N1
A_domestic_duck_England_041295_2022_H5N1
A_chicken_Saratov_29-07V_2021_H5N1
A_Ciconia_ciconia_Spain_22VIR12774-34_3519-1_2022_H5N1
A_goose_Scotland_022633_2022_H5N1
A_chicken_England_138155_2022_H5N1
A_Gallus_gallus_Belgium_3194_0001_2022_H5N1
A_chicken_England_137719_2022_H5N1
A_Great_white_pelican_Israel_123_2022_H5N1
A_chicken_England_125420_2022_H5N1
A_domestic_goose_England_132125_2022_H5N1
A_Egretta_garzetta_Spain_22VIR12774-19_3519-2_2022_H5N1
A_turkey_England_137992_2022_H5N1
A_domestic_duck_England_042266_2022_H5N1
A_Chicken_England_143642_2022_H5N1
A_domestic_goose_England_041268_2022_H5N1
A_Domestic_goose_England_006847_2022_H5N1
A_chicken_England_014330_2022_H5N1
A_Turkey_England_130299_2022_H5N1
A_Mute_Swan_Wales_057697_2022_H5N1
A_goose_Scotland_022634_2022_H5N1
A_chicken_Scotland_024309_2022_H5N1
A_Turkey_England_129898_2022_H5N1
A_chicken_England_118828_2022_H5N1
A_Greylag_goose_Scotland_024915_2022_H5N1
A_Ciconia_ciconia_Spain_22VIR12774-37_3641-1_2022_H5N1
A_duck_France_22P020165_2022_H5N1
A_Branta_canadensis_Belgium_5177_0003_2022_H5N1
A_common_kestel_Israel_49-2_2022_H5N1
A_Common_eider_England_023549_2022_H5N1
A_Larus_canus_Belgium_1668_0019_2022_H5N1
A_fox_England_M0025-12-21_2021_H5N1
A_wild_goose_Spain_22VIR12774-16_3024-6_2022_H5N1
A_Turkey_England_130038_2022_H5N1
A_chicken_England_152082_2022_H5N1
A_Chicken_England_129801_2022_H5N1
A_duck_Egypt_BA20360C_2022_H5N1
A_duck_Egypt_BA20360OP_2022_H5N1
A_turkey_England_138376_2022_H5N1
A_mute_swan_England_253063_2022_H5N1
A_kestrel_England_244611_2021_H5N1
A_Ciconia_ciconia_Spain_22VIR12774-30_3394-2_2022_H5N1
A_pheasant_Wales_016441_2022_H5N1
A_duck_Saratov_29-08V_2021_H5N1
A_chicken_England_131916_2022_H5N1
A_domestic_duck_Scotland_024305_2022_H5N1
A_turkey_England_132310_2022_H5N1
A_Domestic_Duck_England_128223_2022_H5N1
A_Branta_canadensis_Belgium_4821_0001_2022_H5N1
A_Harbour_Seal_Scotland_162919_2022_H5N1
A_Turkey_England_143717_2022_H5N1
A_common_crane_Israel_670_2021_H5N1
A_pheasant_England_019746_2022_H5N1
A_Domestic_Duck_England_134956_2022_H5N1
A_domestic_duck_England_141358_2022_H5N1
A_fox_England_M0083-03-22_2022_H5N1
A_gull_France_22P015977_2022_H5N1
A_fox_France_23P002844_2023_H5N1
A_mallard_22P019377_France_2022_H5N1
A_Anser_anser_France_22P007390_2022_H5N1
A_swan_Netherlands_20017772-002_2020_H5N1
0.002

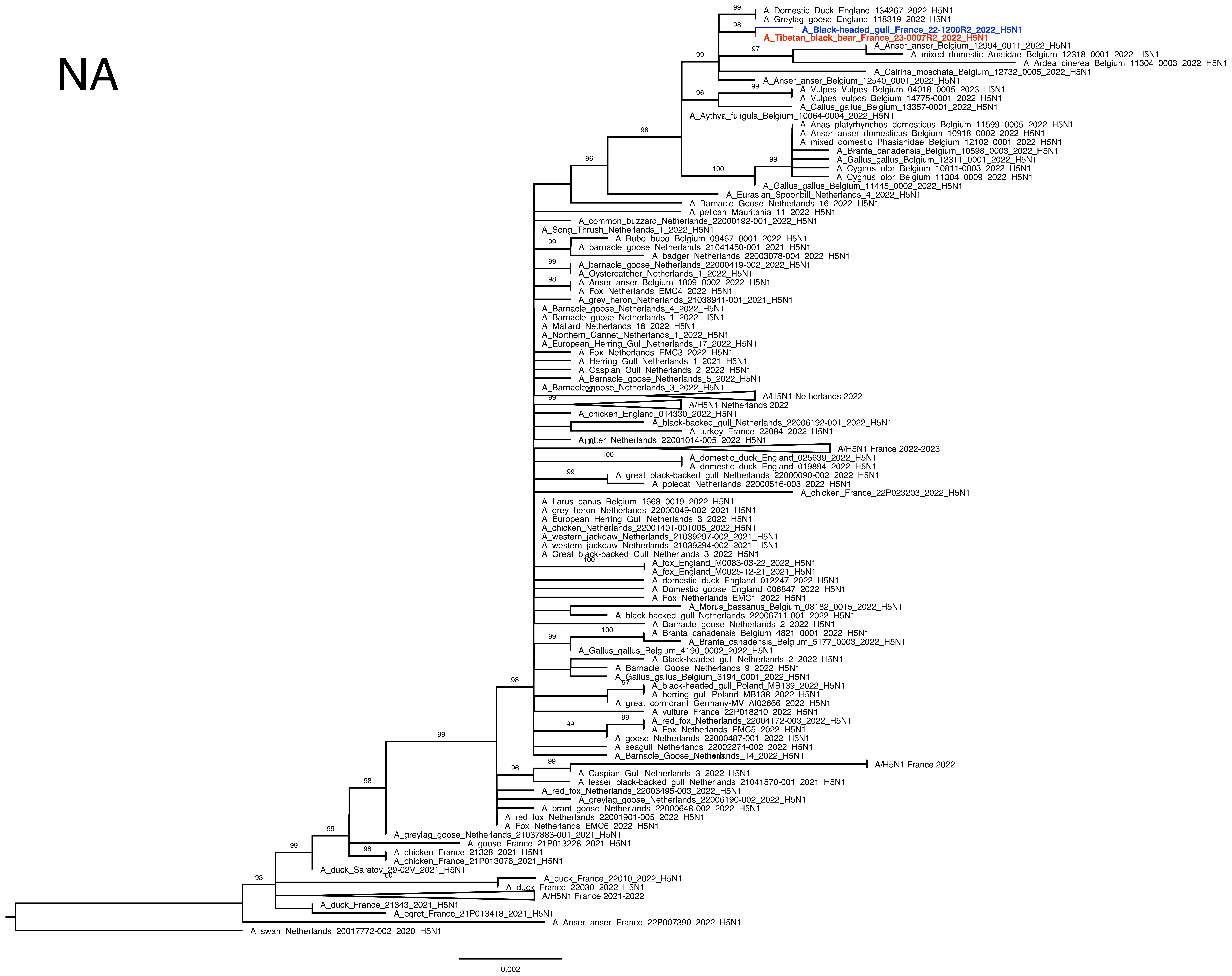
NA
A_Domestic_Duck_England_134267_2022_H5N1
A_Greylag_goose_England_118319_2022_H5N1
A_Black-headed_gull_France_22-1200R2_2022_H5N1
A_Tibetan_black_bear_France_23-0007R2_2022_H5N1
A_Anser_anser_Belgium_12994_0011_2022_H5N1
A_mixed_domestic_Anatidae_Belgium_12318_0001_2022_H5N1
A_Ardea_cinerea_Belgium_11304_0003_2022_H5N1
A_Cairina_moschata_Belgium_12732_0005_2022_H5N1
A_Anser_anser_Belgium_12540_0001_2022_H5N1
A_Vulpes_Vulpes_Belgium_04018_0005_2023_H5N1
A_Vulpes_vulpes_Belgium_14775-0001_2022_H5N1
A_Gallus_gallus_Belgium_13357-0001_2022_H5N1
A_Aythya_fuligula_Belgium_10064-0004_2022_H5N1
A_Anas_platyrhynchos_domesticus_Belgium_11599_0005_2022_H5N1
A_Anser_anser_domesticus_Belgium_10918_0002_2022_H5N1
A_mixed_domestic_Phasianidae_Belgium_12102_0001_2022_H5N1
A_Branta_canadensis_Belgium_10598_0003_2022_H5N1
A_Gallus_gallus_Belgium_12311_0001_2022_H5N1
A_Cygnus_olor_Belgium_10811-0003_2022_H5N1
A_Cygnus_olor_Belgium_11304_0009_2022_H5N1
A_Gallus_gallus_Belgium_11445_0002_2022_H5N1
A_Eurasian_Spoonbill_Netherlands_4_2022_H5N1
A_Barnacle_Goose_Netherlands_16_2022_H5N1
A_pelican_Mauritania_11_2022_H5N1
A_common_buzzard_Netherlands_22000192-001_2022_H5N1
A_Song_Thrush_Netherlands_1_2022_H5N1
A_Bubo_bubo_Belgium_09467_0001_2022_H5N1
A_barnacle_goose_Netherlands_21041450-001_2021_H5N1
A_badger_Netherlands_22003078-004_2022_H5N1
A_barnacle_goose_Netherlands_22000419-002_2022_H5N1
A_Oystercatcher_Netherlands_1_2022_H5N1
A_Anser_anser_Belgium_1809_0002_2022_H5N1
A_Fox_Netherlands_EMC4_2022_H5N1
A_grey_heron_Netherlands_21038941-001_2021_H5N1
A_Barnacle_goose_Netherlands_4_2022_H5N1
A_Barnacle_goose_Netherlands_1_2022_H5N1
A_Mallard_Netherlands_18_2022_H5N1
A_Northern_Gannet_Netherlands_1_2022_H5N1
A_European_Herring_Gull_Netherlands_17_2022_H5N1
A_Fox_Netherlands_EMC3_2022_H5N1
A_Herring_Gull_Netherlands_1_2021_H5N1
A_Caspian_Gull_Netherlands_2_2022_H5N1
A_Barnacle_goose_Netherlands_5_2022_H5N1
A_Barnacle_goose_Netherlands_3_2022_H5N1
A/H5N1 Netherlands 2022
A/H5N1 Netherlands 2022
A_chicken_England_014330_2022_H5N1
A_black-backed_gull_Netherlands_22006192-001_2022_H5N1
A_turkey_France_22084_2022_H5N1
A_otter_Netherlands_22001014-005_2022_H5N1
A/H5N1 France 2022-2023
A_domestic_duck_England_025639_2022_H5N1
A_domestic_duck_England_019894_2022_H5N1
A_great_black-backed_gull_Netherlands_22000090-002_2022_H5N1
A_polecat_Netherlands_22000516-003_2022_H5N1
A_chicken_France_22P023203_2022_H5N1
A_Larus_canus_Belgium_1668_0019_2022_H5N1
A_grey_heron_Netherlands_22000049-002_2021_H5N1
A_European_Herring_Gull_Netherlands_3_2022_H5N1
A_chicken_Netherlands_22001401-001005_2022_H5N1
A_western_jackdaw_Netherlands_21039297-002_2021_H5N1
A_western_jackdaw_Netherlands_21039294-002_2021_H5N1
A_Great_black-backed_Gull_Netherlands_3_2022_H5N1
A_fox_England_M0083-03-22_2022_H5N1
A_fox_England_M0025-12-21_2021_H5N1
A_domestic_duck_England_012247_2022_H5N1
A_Domestic_goose_England_006847_2022_H5N1
A_Fox_Netherlands_EMC1_2022_H5N1
A_Morus_bassanus_Belgium_08182_0015_2022_H5N1
A_black-backed_gull_Netherlands_22006711-001_2022_H5N1
A_Barnacle_goose_Netherlands_2_2022_H5N1
A_Branta_canadensis_Belgium_4821_0001_2022_H5N1
A_Branta_canadensis_Belgium_5177_0003_2022_H5N1
A_Gallus_gallus_Belgium_4190_0002_2022_H5N1
A_Black-headed_gull_Netherlands_2_2022_H5N1
A_Barnacle_Goose_Netherlands_9_2022_H5N1
A_Gallus_gallus_Belgium_3194_0001_2022_H5N1
A_black-headed_gull_Poland_MB139_2022_H5N1
A_herring_gull_Poland_MB138_2022_H5N1
A_great_cormorant_Germany-MV_AI02666_2022_H5N1
A_vulture_France_22P018210_2022_H5N1
A_red_fox_Netherlands_22004172-003_2022_H5N1
A_Fox_Netherlands_EMC5_2022_H5N1
A_goose_Netherlands_22000487-001_2022_H5N1
A_seagull_Netherlands_22002274-002_2022_H5N1
A_Barnacle_Goose_Netherlands_14_2022_H5N1
A/H5N1 France 2022
A_Caspian_Gull_Netherlands_3_2022_H5N1
A_lesser_black-backed_gull_Netherlands_21041570-001_2021_H5N1
A_red_fox_Netherlands_22003495-003_2022_H5N1
A_greylag_goose_Netherlands_22006190-002_2022_H5N1
A_brant_goose_Netherlands_22000648-002_2022_H5N1
A_red_fox_Netherlands_22001901-005_2022_H5N1
A_Fox_Netherlands_EMC6_2022_H5N1
A_greylag_goose_Netherlands_21037883-001_2021_H5N1
A_goose_France_21P013228_2021_H5N1
A_chicken_France_21328_2021_H5N1
A_chicken_France_21P013076_2021_H5N1
A_duck_Saratov_29-02V_2021_H5N1
A_duck_France_22010_2022_H5N1
A_duck_France_22030_2022_H5N1
A/H5N1 France 2021-2022
A_duck_France_21343_2021_H5N1
A_egret_France_21P013418_2021_H5N1
A_Anser_anser_France_22P007390_2022_H5N1
A_swan_Netherlands_20017772-002_2020_H5N1
0.002

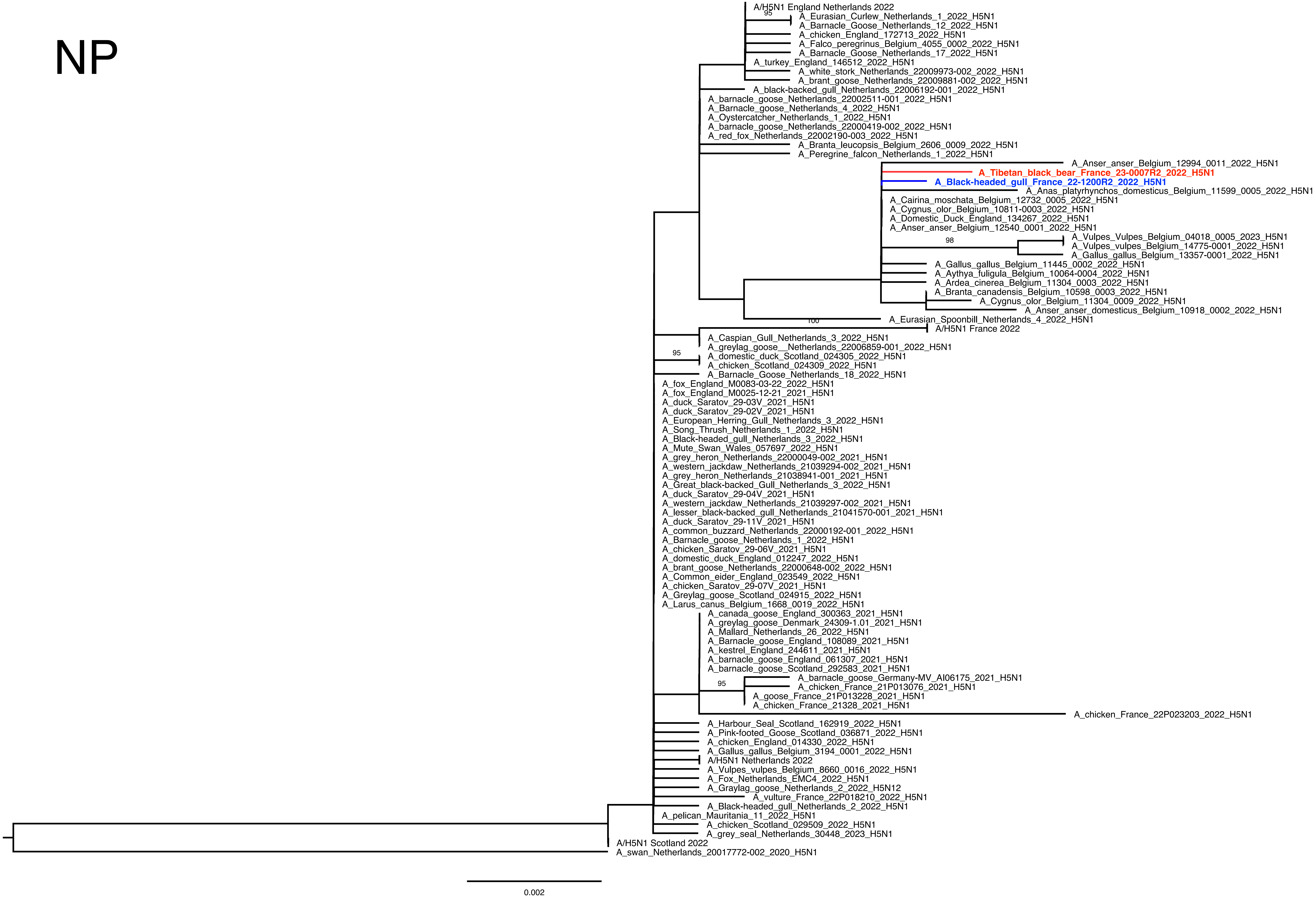
NP
A/H5N1 England Netherlands 2022
95
A_Eurasian_Curlew_Netherlands_1_2022_H5N1
A_Barnacle_Goose_Netherlands_12_2022_H5N1
A_chicken_England_172713_2022_H5N1
A_Falco_peregrinus_Belgium_4055_0002_2022_H5N1
A_Barnacle_Goose_Netherlands_17_2022_H5N1
A_turkey_England_146512_2022_H5N1
A_white_stork_Netherlands_22009973-002_2022_H5N1
A_brant_goose_Netherlands_22009881-002_2022_H5N1
A_black-backed_gull_Netherlands_22006192-001_2022_H5N1
A_barnacle_goose_Netherlands_22002511-001_2022_H5N1
A_Barnacle_goose_Netherlands_4_2022_H5N1
A_Oystercatcher_Netherlands_1_2022_H5N1
A_barnacle_goose_Netherlands_22000419-002_2022_H5N1
A_red_fox_Netherlands_22002190-003_2022_H5N1
A_Branta_leucopsis_Belgium_2606_0009_2022_H5N1
A_Peregrine_falcon_Netherlands_1_2022_H5N1
A_Anser_anser_Belgium_12994_0011_2022_H5N1
A_Tibetan_black_bear_France_23-0007R2_2022_H5N1
A_Black-headed_gull_France_22-1200R2_2022_H5N1
A_Anas_platyrhynchos_domesticus_Belgium_11599_0005_2022_H5N1
A_Cairina_moschata_Belgium_12732_0005_2022_H5N1
A_Cygnus_olor_Belgium_10811-0003_2022_H5N1
A_Domestic_Duck_England_134267_2022_H5N1
A_Anser_anser_Belgium_12540_0001_2022_H5N1
A_Vulpes_Vulpes_Belgium_04018_0005_2023_H5N1
98
A_Vulpes_vulpes_Belgium_14775-0001_2022_H5N1
A_Gallus_gallus_Belgium_13357-0001_2022_H5N1
A_Gallus_gallus_Belgium_11445_0002_2022_H5N1
A_Aythya_fuligula_Belgium_10064-0004_2022_H5N1
A_Ardea_cinerea_Belgium_11304_0003_2022_H5N1
A_Branta_canadensis_Belgium_10598_0003_2022_H5N1
A_Cygnus_olor_Belgium_11304_0009_2022_H5N1
A_Anser_anser_domesticus_Belgium_10918_0002_2022_H5N1
100
A_Eurasian_Spoonbill_Netherlands_4_2022_H5N1
A/H5N1 France 2022
A_Caspian_Gull_Netherlands_3_2022_H5N1
A_greylag_goose__Netherlands_22006859-001_2022_H5N1
95
A_domestic_duck_Scotland_024305_2022_H5N1
A_chicken_Scotland_024309_2022_H5N1
A_Barnacle_Goose_Netherlands_18_2022_H5N1
A_fox_England_M0083-03-22_2022_H5N1
A_fox_England_M0025-12-21_2021_H5N1
A_duck_Saratov_29-03V_2021_H5N1
A_duck_Saratov_29-02V_2021_H5N1
A_European_Herring_Gull_Netherlands_3_2022_H5N1
A_Song_Thrush_Netherlands_1_2022_H5N1
A_Black-headed_gull_Netherlands_3_2022_H5N1
A_Mute_Swan_Wales_057697_2022_H5N1
A_grey_heron_Netherlands_22000049-002_2021_H5N1
A_western_jackdaw_Netherlands_21039294-002_2021_H5N1
A_grey_heron_Netherlands_21038941-001_2021_H5N1
A_Great_black-backed_Gull_Netherlands_3_2022_H5N1
A_duck_Saratov_29-04V_2021_H5N1
A_western_jackdaw_Netherlands_21039297-002_2021_H5N1
A_lesser_black-backed_gull_Netherlands_21041570-001_2021_H5N1
A_duck_Saratov_29-11V_2021_H5N1
A_common_buzzard_Netherlands_22000192-001_2022_H5N1
A_Barnacle_goose_Netherlands_1_2022_H5N1
A_chicken_Saratov_29-06V_2021_H5N1
A_domestic_duck_England_012247_2022_H5N1
A_brant_goose_Netherlands_22000648-002_2022_H5N1
A_Common_eider_England_023549_2022_H5N1
A_chicken_Saratov_29-07V_2021_H5N1
A_Greylag_goose_Scotland_024915_2022_H5N1
A_Larus_canus_Belgium_1668_0019_2022_H5N1
A_canada_goose_England_300363_2021_H5N1
A_greylag_goose_Denmark_24309-1.01_2021_H5N1
A_Mallard_Netherlands_26_2022_H5N1
A_Barnacle_goose_England_108089_2021_H5N1
A_kestrel_England_244611_2021_H5N1
A_barnacle_goose_England_061307_2021_H5N1
A_barnacle_goose_Scotland_292583_2021_H5N1
A_barnacle_goose_Germany-MV_AI06175_2021_H5N1
95
A_chicken_France_21P013076_2021_H5N1
A_goose_France_21P013228_2021_H5N1
A_chicken_France_21328_2021_H5N1
A_chicken_France_22P023203_2022_H5N1
A_Harbour_Seal_Scotland_162919_2022_H5N1
A_Pink-footed_Goose_Scotland_036871_2022_H5N1
A_chicken_England_014330_2022_H5N1
A_Gallus_gallus_Belgium_3194_0001_2022_H5N1
A/H5N1 Netherlands 2022
A_Vulpes_vulpes_Belgium_8660_0016_2022_H5N1
A_Fox_Netherlands_EMC4_2022_H5N1
A_Graylag_goose_Netherlands_2_2022_H5N12
A_vulture_France_22P018210_2022_H5N1
A_Black-headed_gull_Netherlands_2_2022_H5N1
A_pelican_Mauritania_11_2022_H5N1
A_chicken_Scotland_029509_2022_H5N1
A_grey_seal_Netherlands_30448_2023_H5N1
A/H5N1 Scotland 2022
A_swan_Netherlands_20017772-002_2020_H5N1
0.002

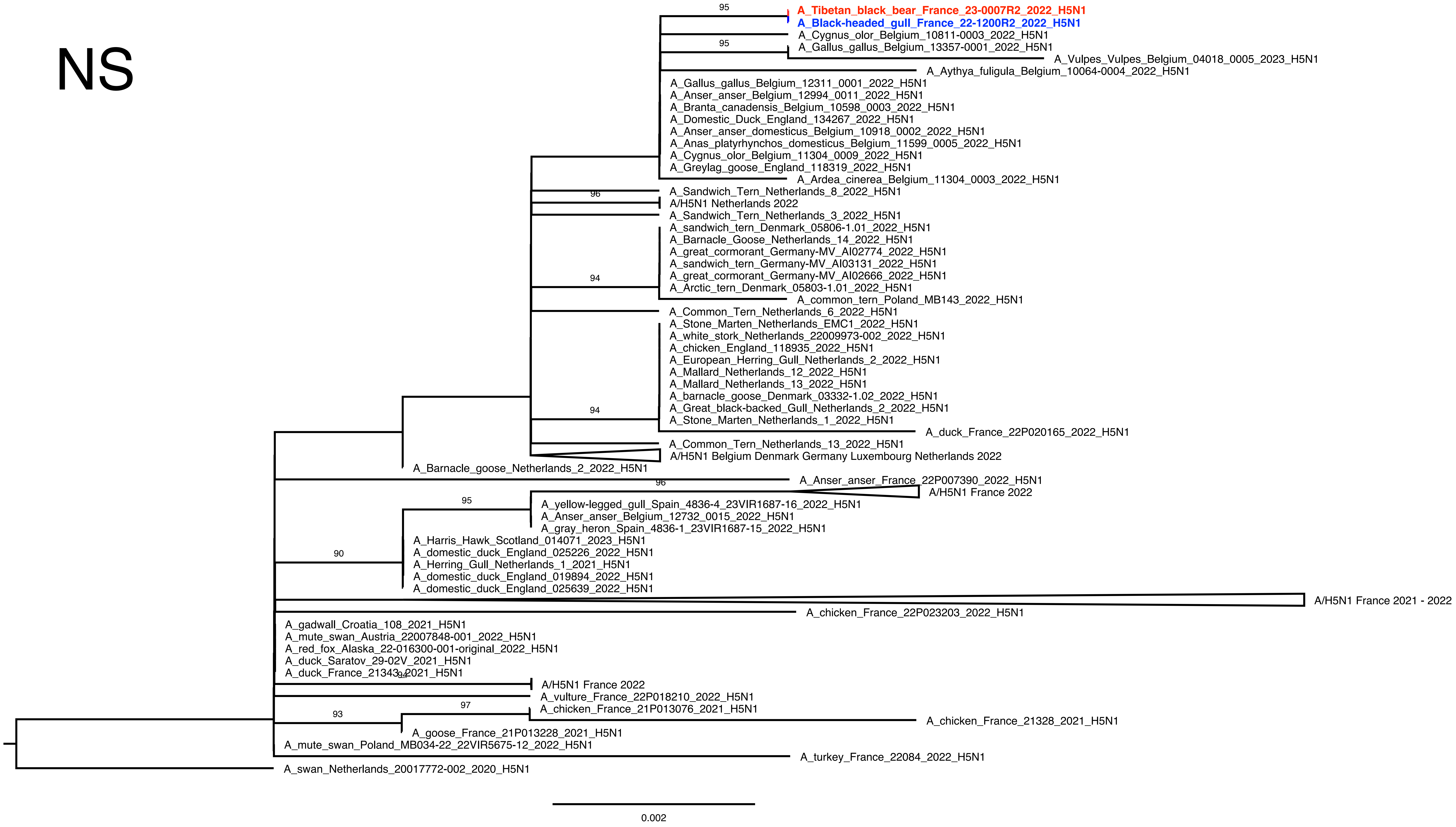
NS
A_Tibetan_black_bear_France_23-0007R2_2022_H5N1
A_Black-headed_gull_France_22-1200R2_2022_H5N1
A_Cygnus_olor_Belgium_10811-0003_2022_H5N1
A_Gallus_gallus_Belgium_13357-0001_2022_H5N1
A_Vulpes_Vulpes_Belgium_04018_0005_2023_H5N1
A_Aythya_fuligula_Belgium_10064-0004_2022_H5N1
A_Gallus_gallus_Belgium_12311_0001_2022_H5N1
A_Anser_anser_Belgium_12994_0011_2022_H5N1
A_Branta_canadensis_Belgium_10598_0003_2022_H5N1
A_Domestic_Duck_England_134267_2022_H5N1
A_Anser_anser_domesticus_Belgium_10918_0002_2022_H5N1
A_Anas_platyrhynchos_domesticus_Belgium_11599_0005_2022_H5N1
A_Cygnus_olor_Belgium_11304_0009_2022_H5N1
A_Greylag_goose_England_118319_2022_H5N1
A_Ardea_cinerea_Belgium_11304_0003_2022_H5N1
A_Sandwich_Tern_Netherlands_8_2022_H5N1
A/H5N1 Netherlands 2022
A_Sandwich_Tern_Netherlands_3_2022_H5N1
A_sandwich_tern_Denmark_05806-1.01_2022_H5N1
A_Barnacle_Goose_Netherlands_14_2022_H5N1
A_great_cormorant_Germany-MV_AI02774_2022_H5N1
A_sandwich_tern_Germany-MV_AI03131_2022_H5N1
A_great_cormorant_Germany-MV_AI02666_2022_H5N1
A_Arctic_tern_Denmark_05803-1.01_2022_H5N1
A_common_tern_Poland_MB143_2022_H5N1
A_Common_Tern_Netherlands_6_2022_H5N1
A_Stone_Marten_Netherlands_EMC1_2022_H5N1
A_white_stork_Netherlands_22009973-002_2022_H5N1
A_chicken_England_118935_2022_H5N1
A_European_Herring_Gull_Netherlands_2_2022_H5N1
A_Mallard_Netherlands_12_2022_H5N1
A_Mallard_Netherlands_13_2022_H5N1
A_barnacle_goose_Denmark_03332-1.02_2022_H5N1
A_Great_black-backed_Gull_Netherlands_2_2022_H5N1
A_Stone_Marten_Netherlands_1_2022_H5N1
A_duck_France_22P020165_2022_H5N1
A_Common_Tern_Netherlands_13_2022_H5N1
A/H5N1 Belgium Denmark Germany Luxembourg Netherlands 2022
A_Barnacle_goose_Netherlands_2_2022_H5N1
A_Anser_anser_France_22P007390_2022_H5N1
A/H5N1 France 2022
A_yellow-legged_gull_Spain_4836-4_23VIR1687-16_2022_H5N1
A_Anser_anser_Belgium_12732_0015_2022_H5N1
A_gray_heron_Spain_4836-1_23VIR1687-15_2022_H5N1
A_Harris_Hawk_Scotland_014071_2023_H5N1
A_domestic_duck_England_025226_2022_H5N1
A_Herring_Gull_Netherlands_1_2021_H5N1
A_domestic_duck_England_019894_2022_H5N1
A_domestic_duck_England_025639_2022_H5N1
A/H5N1 France 2021 - 2022
A_chicken_France_22P023203_2022_H5N1
A_gadwall_Croatia_108_2021_H5N1
A_mute_swan_Austria_22007848-001_2022_H5N1
A_red_fox_Alaska_22-016300-001-original_2022_H5N1
A_duck_Saratov_29-02V_2021_H5N1
A_duck_France_21343_2021_H5N1
A/H5N1 France 2022
A_vulture_France_22P018210_2022_H5N1
A_chicken_France_21P013076_2021_H5N1
A_chicken_France_21328_2021_H5N1
A_goose_France_21P013228_2021_H5N1
A_mute_swan_Poland_MB034-22_22VIR5675-12_2022_H5N1
A_turkey_France_22084_2022_H5N1
A_swan_Netherlands_20017772-002_2020_H5N1
0.002

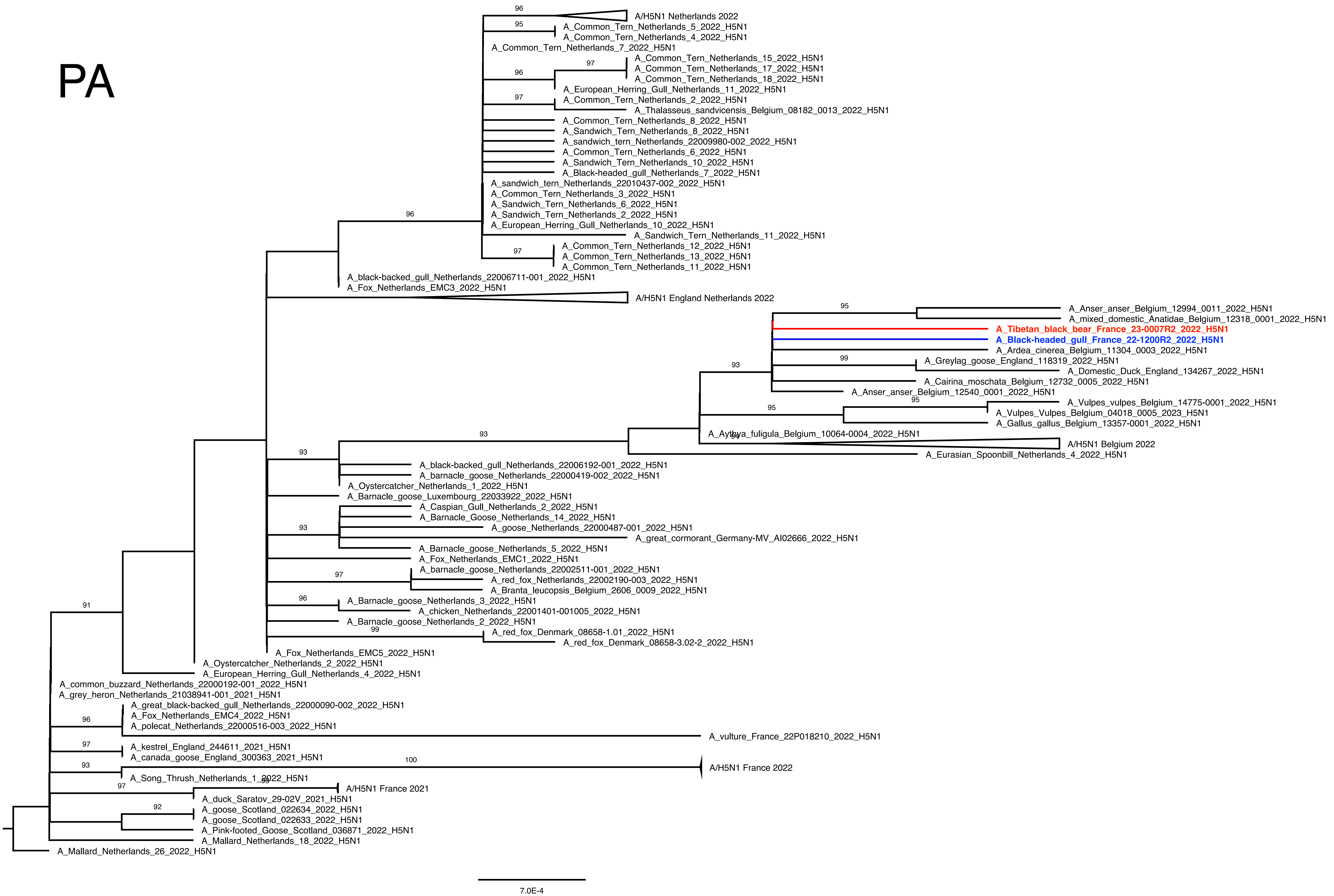
PA
A/H5N1 Netherlands 2022
A_Common_Tern_Netherlands_5_2022_H5N1
A_Common_Tern_Netherlands_4_2022_H5N1
A_Common_Tern_Netherlands_7_2022_H5N1
A_Common_Tern_Netherlands_15_2022_H5N1
A_Common_Tern_Netherlands_17_2022_H5N1
A_Common_Tern_Netherlands_18_2022_H5N1
A_European_Herring_Gull_Netherlands_11_2022_H5N1
A_Common_Tern_Netherlands_2_2022_H5N1
A_Thalasseus_sandvicensis_Belgium_08182_0013_2022_H5N1
A_Common_Tern_Netherlands_8_2022_H5N1
A_Sandwich_Tern_Netherlands_8_2022_H5N1
A_sandwich_tern_Netherlands_22009980-002_2022_H5N1
A_Common_Tern_Netherlands_6_2022_H5N1
A_Sandwich_Tern_Netherlands_10_2022_H5N1
A_Black-headed_gull_Netherlands_7_2022_H5N1
A_sandwich_tern_Netherlands_22010437-002_2022_H5N1
A_Common_Tern_Netherlands_3_2022_H5N1
A_Sandwich_Tern_Netherlands_6_2022_H5N1
A_Sandwich_Tern_Netherlands_2_2022_H5N1
A_European_Herring_Gull_Netherlands_10_2022_H5N1
A_Sandwich_Tern_Netherlands_11_2022_H5N1
A_Common_Tern_Netherlands_12_2022_H5N1
A_Common_Tern_Netherlands_13_2022_H5N1
A_Common_Tern_Netherlands_11_2022_H5N1
A_black-backed_gull_Netherlands_22006711-001_2022_H5N1
A_Fox_Netherlands_EMC3_2022_H5N1
A/H5N1 England Netherlands 2022
A_Anser_anser_Belgium_12994_0011_2022_H5N1
A_mixed_domestic_Anatidae_Belgium_12318_0001_2022_H5N1
A_Tibetan_black_bear_France_23-0007R2_2022_H5N1
A_Black-headed_gull_France_22-1200R2_2022_H5N1
A_Ardea_cinerea_Belgium_11304_0003_2022_H5N1
A_Greylag_goose_England_118319_2022_H5N1
A_Domestic_Duck_England_134267_2022_H5N1
A_Cairina_moschata_Belgium_12732_0005_2022_H5N1
A_Anser_anser_Belgium_12540_0001_2022_H5N1
A_Vulpes_vulpes_Belgium_14775-0001_2022_H5N1
A_Vulpes_Vulpes_Belgium_04018_0005_2023_H5N1
A_Gallus_gallus_Belgium_13357-0001_2022_H5N1
A_Aythya_fuligula_Belgium_10064-0004_2022_H5N1
A/H5N1 Belgium 2022
A_Eurasian_Spoonbill_Netherlands_4_2022_H5N1
A_black-backed_gull_Netherlands_22006192-001_2022_H5N1
A_barnacle_goose_Netherlands_22000419-002_2022_H5N1
A_Oystercatcher_Netherlands_1_2022_H5N1
A_Barnacle_goose_Luxembourg_22033922_2022_H5N1
A_Caspian_Gull_Netherlands_2_2022_H5N1
A_Barnacle_Goose_Netherlands_14_2022_H5N1
A_goose_Netherlands_22000487-001_2022_H5N1
A_great_cormorant_Germany-MV_AI02666_2022_H5N1
A_Barnacle_goose_Netherlands_5_2022_H5N1
A_Fox_Netherlands_EMC1_2022_H5N1
A_barnacle_goose_Netherlands_22002511-001_2022_H5N1
A_red_fox_Netherlands_22002190-003_2022_H5N1
A_Branta_leucopsis_Belgium_2606_0009_2022_H5N1
A_Barnacle_goose_Netherlands_3_2022_H5N1
A_chicken_Netherlands_22001401-001005_2022_H5N1
A_Barnacle_goose_Netherlands_2_2022_H5N1
A_red_fox_Denmark_08658-1.01_2022_H5N1
A_red_fox_Denmark_08658-3.02-2_2022_H5N1
A_Fox_Netherlands_EMC5_2022_H5N1
A_Oystercatcher_Netherlands_2_2022_H5N1
A_European_Herring_Gull_Netherlands_4_2022_H5N1
A_common_buzzard_Netherlands_22000192-001_2022_H5N1
A_grey_heron_Netherlands_21038941-001_2021_H5N1
A_great_black-backed_gull_Netherlands_22000090-002_2022_H5N1
A_Fox_Netherlands_EMC4_2022_H5N1
A_polecat_Netherlands_22000516-003_2022_H5N1
A_vulture_France_22P018210_2022_H5N1
A_kestrel_England_244611_2021_H5N1
A_canada_goose_England_300363_2021_H5N1
A/H5N1 France 2022
A_Song_Thrush_Netherlands_1_2022_H5N1
A/H5N1 France 2021
A_duck_Saratov_29-02V_2021_H5N1
A_goose_Scotland_022634_2022_H5N1
A_goose_Scotland_022633_2022_H5N1
A_Pink-footed_Goose_Scotland_036871_2022_H5N1
A_Mallard_Netherlands_18_2022_H5N1
A_Mallard_Netherlands_26_2022_H5N1
7.0E-4

PB1

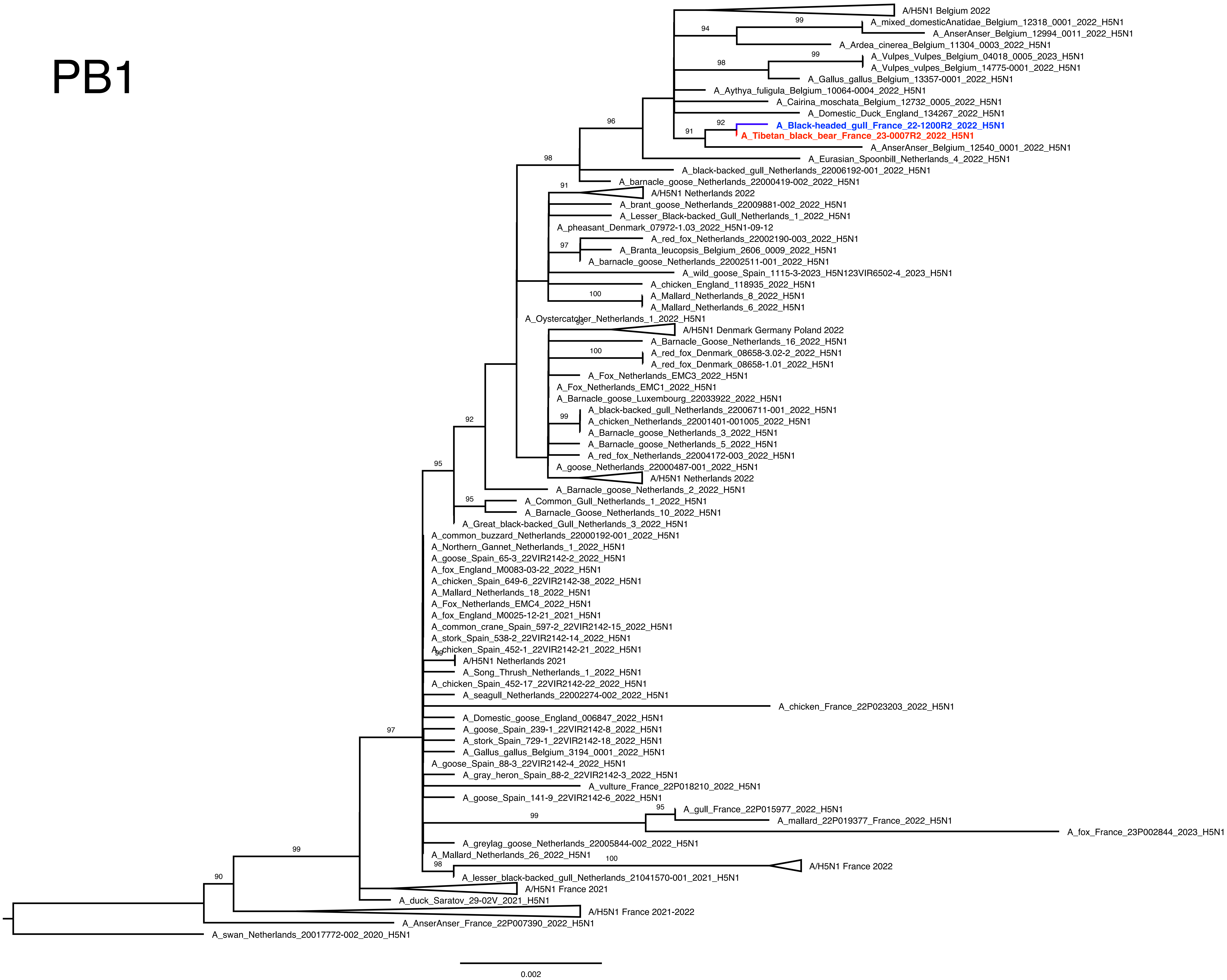
A/H5N1 Belgium 2022
A_mixed_domesticAnatidae_Belgium_12318_0001_2022_H5N1
A_AnserAnser_Belgium_12994_0011_2022_H5N1
A_Ardea_cinerea_Belgium_11304_0003_2022_H5N1
A_Vulpes_Vulpes_Belgium_04018_0005_2023_H5N1
A_Vulpes_vulpes_Belgium_14775-0001_2022_H5N1
A_Gallus_gallus_Belgium_13357-0001_2022_H5N1
A_Aythya_fuligula_Belgium_10064-0004_2022_H5N1
A_Cairina_moschata_Belgium_12732_0005_2022_H5N1
A_Domestic_Duck_England_134267_2022_H5N1
A_Black-headed_gull_France_22-1200R2_2022_H5N1
A_Tibetan_black_bear_France_23-0007R2_2022_H5N1
A_AnserAnser_Belgium_12540_0001_2022_H5N1
A_Eurasian_Spoonbill_Netherlands_4_2022_H5N1
A_black-backed_gull_Netherlands_22006192-001_2022_H5N1
A_barnacle_goose_Netherlands_22000419-002_2022_H5N1
A/H5N1 Netherlands 2022
A_brant_goose_Netherlands_22009881-002_2022_H5N1
A_Lesser_Black-backed_Gull_Netherlands_1_2022_H5N1
A_pheasant_Denmark_07972-1.03_2022_H5N1-09-12
A_red_fox_Netherlands_22002190-003_2022_H5N1
A_Branta_leucopsis_Belgium_2606_0009_2022_H5N1
A_barnacle_goose_Netherlands_22002511-001_2022_H5N1
A_wild_goose_Spain_1115-3-2023_H5N123VIR6502-4_2023_H5N1
A_chicken_England_118935_2022_H5N1
A_Mallard_Netherlands_8_2022_H5N1
A_Mallard_Netherlands_6_2022_H5N1
A_Oystercatcher_Netherlands_1_2022_H5N1
A/H5N1 Denmark Germany Poland 2022
A_Barnacle_Goose_Netherlands_16_2022_H5N1
A_red_fox_Denmark_08658-3.02-2_2022_H5N1
A_red_fox_Denmark_08658-1.01_2022_H5N1
A_Fox_Netherlands_EMC3_2022_H5N1
A_Fox_Netherlands_EMC1_2022_H5N1
A_Barnacle_goose_Luxembourg_22033922_2022_H5N1
A_black-backed_gull_Netherlands_22006711-001_2022_H5N1
A_chicken_Netherlands_22001401-001005_2022_H5N1
A_Barnacle_goose_Netherlands_3_2022_H5N1
A_Barnacle_goose_Netherlands_5_2022_H5N1
A_red_fox_Netherlands_22004172-003_2022_H5N1
A_goose_Netherlands_22000487-001_2022_H5N1
A/H5N1 Netherlands 2022
A_Barnacle_goose_Netherlands_2_2022_H5N1
A_Common_Gull_Netherlands_1_2022_H5N1
A_Barnacle_Goose_Netherlands_10_2022_H5N1
A_Great_black-backed_Gull_Netherlands_3_2022_H5N1
A_common_buzzard_Netherlands_22000192-001_2022_H5N1
A_Northern_Gannet_Netherlands_1_2022_H5N1
A_goose_Spain_65-3_22VIR2142-2_2022_H5N1
A_fox_England_M0083-03-22_2022_H5N1
A_chicken_Spain_649-6_22VIR2142-38_2022_H5N1
A_Mallard_Netherlands_18_2022_H5N1
A_Fox_Netherlands_EMC4_2022_H5N1
A_fox_England_M0025-12-21_2021_H5N1
A_common_crane_Spain_597-2_22VIR2142-15_2022_H5N1
A_stork_Spain_538-2_22VIR2142-14_2022_H5N1
A_chicken_Spain_452-1_22VIR2142-21_2022_H5N1
A/H5N1 Netherlands 2021
A_Song_Thrush_Netherlands_1_2022_H5N1
A_chicken_Spain_452-17_22VIR2142-22_2022_H5N1
A_seagull_Netherlands_22002274-002_2022_H5N1
A_chicken_France_22P023203_2022_H5N1
A_Domestic_goose_England_006847_2022_H5N1
A_goose_Spain_239-1_22VIR2142-8_2022_H5N1
A_stork_Spain_729-1_22VIR2142-18_2022_H5N1
A_Gallus_gallus_Belgium_3194_0001_2022_H5N1
A_goose_Spain_88-3_22VIR2142-4_2022_H5N1
A_gray_heron_Spain_88-2_22VIR2142-3_2022_H5N1
A_vulture_France_22P018210_2022_H5N1
A_goose_Spain_141-9_22VIR2142-6_2022_H5N1
A_gull_France_22P015977_2022_H5N1
A_mallard_22P019377_France_2022_H5N1
A_fox_France_23P002844_2023_H5N1
A_greylag_goose_Netherlands_22005844-002_2022_H5N1
A_Mallard_Netherlands_26_2022_H5N1
A/H5N1 France 2022
A_lesser_black-backed_gull_Netherlands_21041570-001_2021_H5N1
A/H5N1 France 2021
A_duck_Saratov_29-02V_2021_H5N1
A/H5N1 France 2021-2022
A_AnserAnser_France_22P007390_2022_H5N1
A_swan_Netherlands_20017772-002_2020_H5N1
0.002
