# Supplementary material for: High pathogenicity avian influenza A (H5N1) clade 2.3.4.4b virus infection in a captive Tibetan black bear (Ursus thibetanus): investigations based on paraffin-embedded tissues, France, 2022
Source: Microbiol Spectr. 2024 Feb 2;12(3):e03736-23. doi: 10.1128/spectrum.03736-23 (PMC10913436; doi:10.1128/spectrum.03736-23)
Supplement: Tables S1 to S3 — Summary of animals affected by the epizootic, and subtissular localization of viral antigen/RNA in the tissues. [file spectrum.03736-23-s0002.docx]

**Supplementary Table 1:** Summary of animals affected by the epizootic.

* Animals with only suspected H5N1 infection (no diagnostic test performed).

| **Species** | **Onset of clinical signs** | **Clinical signs** | **Date of death** | **Location** |
| --- | --- | --- | --- | --- |
| *Ursus thibetanus* | 31/10/2022 | Hyperthermia, depression, severe dyspnea, diarrhea | 01/11/2022 | Bear enclosure |
| *Pelecanus rufescens* | Unknown | Unknown | 04/11/2022 | Next to the bear enclosure |
| *Ursus thibetanus** | 05/11/2022 | Coughing, nasal discharge | (Recovery) | Bear enclosure |
| *Ursus thibetanus** | 07/11/2022 | Cough, depression, mild dyspnea | (Recovery) | Bear enclosure |
| *Pelecanus rufescens* | Unknown | Unknown | 14/11/2022 | Elsewhere in the zoo |
| *Chroicocephalus ridibundus* | Unknown | Unknown | 14/11/2022 | Bear enclosure |
| *Coloeus monedula* | Unknown | Unknown | 15/11/2022 | Elsewhere in the zoo |
| *Ursus thibetanus** | 16/11/2022 | Cough, depression, mild dyspnea | (Recovery) | Bear enclosure |
| *Ursus thibetanus** | 27/11/2022 | Cough, depression, mild dyspnea | (Recovery) | Bear enclosure |

**Supplementary Table 2:** Subtissular localization of viral antigen/RNA in the tissues obtained from the Tibetan black bear naturally-infected with H5N1 clade 2.3.4.4b HPAIV.

|  | **Subtissular detection** | | |
| --- | --- | --- | --- |
| **Organ** | **Immunohistochemistry targeting IAV nucleoprotein** | **RNAscope *In situ* Hybridization targeting AIAV M gene RNAs** |  |
| **Lymph node** | Leucocytes, perivascular tissue adjacent to foci of fibrinoid necrosis. | Leucocytes, perivascular tissue adjacent to foci of fibrinoid necrosis. |  |
| **Trachea** | Non-specific background staining. | Mesenchymal cells from submucosa and lamina propria, rare endothelial cells. |  |
| **Lung** | Luminal debris, perivascular interlobular mesenchymal cells.  Diffuse non-specific background staining, including endogenous and formalin pigments. | Luminal debris, perivascular interlobular mesenchymal cells, subpleural mesenchymal cells, epithelial cells from alveolar septa, presumptive bronchiolar epithelial cells and luminal debris, mesothelial cells. |  |
| **Heart** | Non-specific background staining. | Mesenchymal cells from myocardial endomysium and perimysium. |  |
| **Spleen** | Non-specific background staining, including endogenous and formalin pigments. | Splenic red pulp (undetermined specific cellular type). |  |
| **Liver** | Non-specific background staining, including endogenous and intraphagocytic pigments. | Negative |  |
| **Stomach** | Non-specific background staining, rare mesenchymal cells from subserosal connective tissue. | Rare mesenchymal cells from subserosal connective tissue. |  |
| **Intestine** | Myenteric plexus nerve trunk and connective tissue, cell debris from superficial lamina propria. | Myenteric plexus nerve trunk and peripheral connective tissue, rare intraluminal cell debris |  |
| **Kidney** | Glomeruli: mesangial cells, rare endothelial cell from capillary loop. | Negative |  |

**Supplementary Table 3:** Subtissular localization of viral antigen/RNA in the tissues obtained from the black-headed gull naturally infected with H5N1 clade 2.3.4.4b HPAIV

|  | **Subtissular detection** | |
| --- | --- | --- |
|  | **Immunohistochemistry targeting IAV nucleoprotein** | **RNAscope *In situ* Hybridization targeting AIAV M gene RNAs** |
| **Brain  (optic lobe, cerebellum)** | Neurons, Purkinje cells, glial cells, neuropil | Neurons, Purkinje cells, glial cells, neuropil |
| **Kidney** | Tubular renal nephrocytes | Nervous ganglion: neurons, glial cells.  Tubular renal nephrocytes |
| **Intestine** | Negative | Negative |
