# Supplementary material for: High pathogenicity avian influenza A (H5N1) clade 2.3.4.4b virus infection in a captive Tibetan black bear (Ursus thibetanus): investigations based on paraffin-embedded tissues, France, 2022
Source: Microbiol Spectr. 2024 Feb 2;12(3):e03736-23. doi: 10.1128/spectrum.03736-23 (PMC10913436; doi:10.1128/spectrum.03736-23)
Supplement: Supplemental file 1 — Bear blood analysis results. [file spectrum.03736-23-s0003.pdf]

Supplemental File 1: bear blood analysis results.
Analysis was performed by the zoo veterinarian prior to the bear death.

Complete blood count:
Red blood cells: 8.01 x 10^{12}/L
Hematocrit: 4.8%
Hemoglobin: 17.6g/dL
Mean corpuscular volume: 59.7fL
Mean corpuscular hemoglobin: 21.9pg
Mean corpuscular hemoglobin concentration: 36.7g/dL
Red cell distribution width: 16.1%
% Reticulocyte: 16.1%
Reticulocytes: 31,6 K/µL
White blood cells: 1.80 x 10^{9}/L
- Neutrophiles: 37.2%
- Lymphocytes: 56.0%
- Monocytes: 2.9%
- Eosinophiles: 3.9%
- Basophiles: 0%

Platelets: 36 K/µL

Blood biochemistry:
Glucose: 1.52g/L
Creatinine: 60mg/L
Urea: 0.436g/L
Total protein: 83g/L
Albumin: 39g/L
Globulin: 44g/L
Alanine transaminase: 225U/L
Alkaline phosphatase: 41U/L
C reactive protein: < 1mg/L
